# The Need for Explicit Teaching of Learning Strategies in Undergraduate Medical Education in Pakistan

**DOI:** 10.12669/pjms.42.2.14398

**Published:** 2026-02

**Authors:** Numera Yousuf, Muhammad Yousuf Ali

**Affiliations:** 1 Numera Yousuf, MBBS Department of Forensic Medicine, Jinnah Sindh Medical University, Karachi, Pakistan; 2 Muhammad Yousuf Ali, BDS Department of Dental Materials, Karachi Metropolitan University, Karachi, Pakistan

Starting university requires students to adjust quickly to new academic demands. One important way to support this transition is by helping students develop strong self-regulated learning skills. Learning strategies are an essential part of self-regulated learning. While structured programs that teach learning strategies have been increasingly integrated into the curricula of many developed countries, such as Canada, the Netherlands, and the United States, this component remains largely absent in medical education curricula in Pakistan. In Pakistan, medical curricula continue to rely on students’ independent development of these skills, highlighting a significant gap in supporting learners during their critical transition into university and clinical training. This gap could be bridged through workshops, short courses or interactive seminars specifically designed to teach learning strategies.

Learning strategies refer to the methods students use to understand, process, and retain information, such as practice testing, spaced repetition, and self-explanation.^1^ These strategies are generally classified as surface, deep, or strategic approaches.^2^ Surface learning strategies focus mainly on mere memorization, whereas, deep learning strategies emphasize on understanding and learning in a holistic way. On the other hand, strategic learning involves organizing one’s study approach to achieve desired outcomes and may involve both surface and deep strategies. A major challenge is that when students don’t truly understand a concept, they find it difficult to use that knowledge in new or unfamiliar situations. This is why choosing the right learning strategies matters so much. Effective strategies help students build understanding that lasts and can be applied when it really counts.

Evidence supports that explicit teaching of effective learning strategies (often a blend of deep and strategic approaches) results in dramatic improvement in academic performance of students.^3^ Yet, many students are unaware of what are effective learning strategies.^3^ As a result, information is often forgotten quickly, forcing students to spend additional time and effort re-learning material. By integrating these strategies into the curriculum, we can equip students not only with medical knowledge but also with the cognitive skills needed to solve complex problems and adapt to changing professional demands. In this way, we can create a learning environment where no student is left behind.

Therefore, we encourage curriculum designers and medical educators to make the explicit teaching of learning strategies an essential component of the early undergraduate years. The teaching programs could include declarative and conditional knowledge of the effective learning strategies along with supporting practice to use them. By doing so, it is expected, that students would be able to better manage their own learning and cope with the challenges of medical training more effectively.
